# Supplementary material for: Interdomain interactions regulate the localization of a lipid transfer protein at ER-PM contact sites
Source: Biol Open. 2021 Mar 18;10(3):bio057422. doi: 10.1242/bio.057422 (PMC7990853; doi:10.1242/bio.057422)
Supplement: Supplementary information [file biolopen-10-057422-s1.pdf]

A.

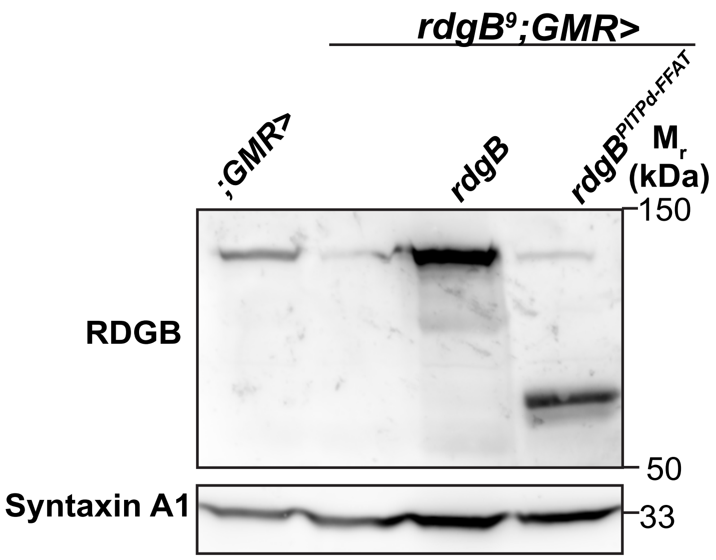

B.

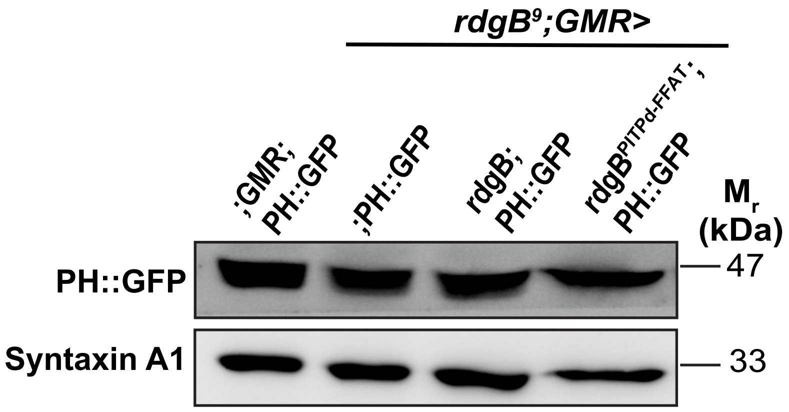

Basak et al., Supplemental Data 1

Figure S1.

A. Western blot of protein extracts made from fly heads of $RDGB^{PITPd\text{-}FFAT}$ and relevant controls. The blot is probed with antibody to RDGB. Syntaxin A1 is used as a loading control (N=3).

B. Western blot of protein extracts made from fly heads of $RDGB^{PITPd\text{-}FFAT}$ and relevant controls expressing PH-PLCδ::GFP probe. The blot is probed with antibody to GFP. Syntaxin A1 is used as a loading control (N=3).

Biology Open • Supplementary information

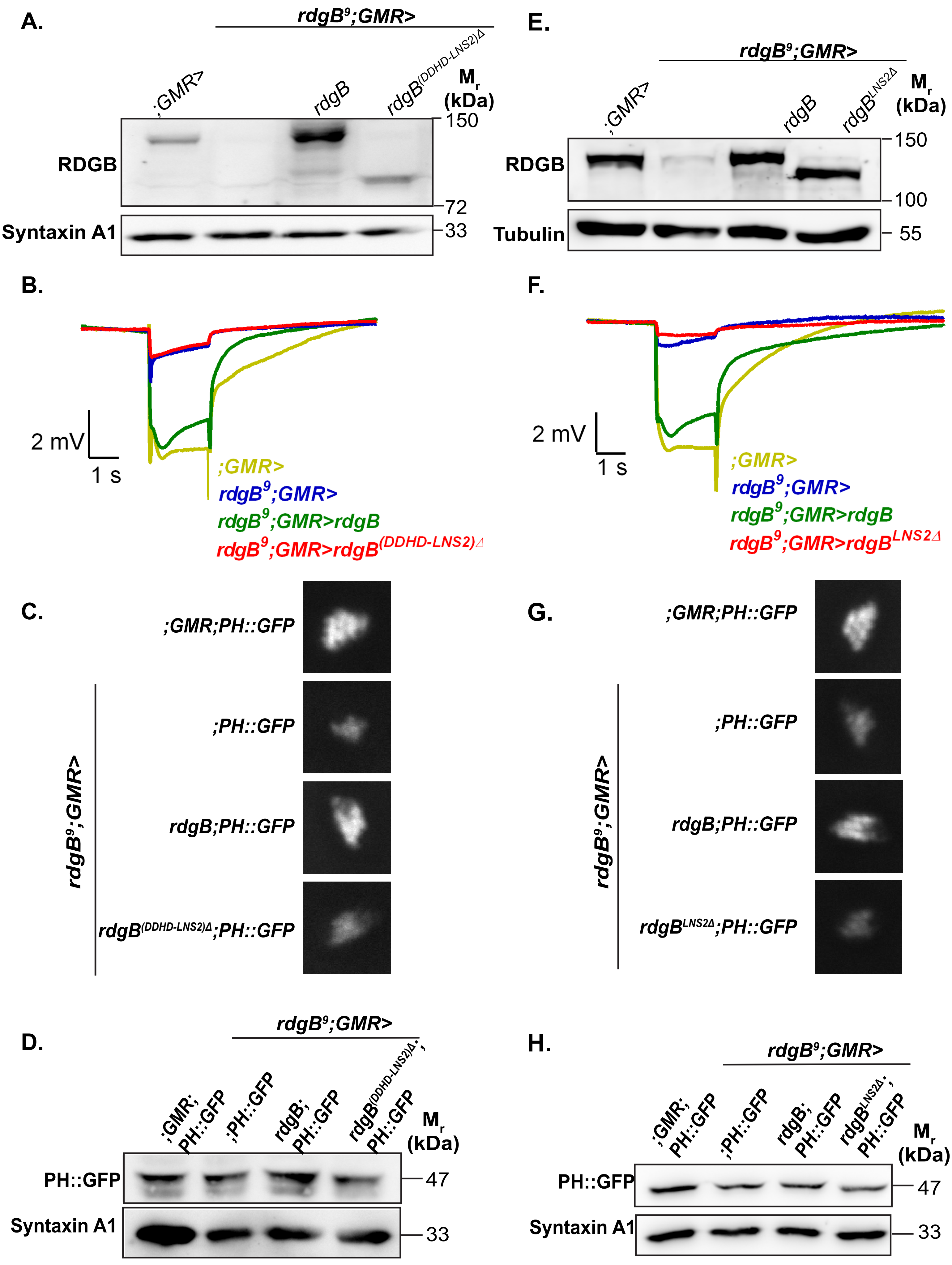

Basak et al., Supplemental Data 2

Figure S2.

A. Western blot of protein extracts made from fly heads of RDGB$^{(DDHD-LNS2)\Delta}$ and relevant controls. The blot is probed with antibody to RDGB. Syntaxin A1 is used as a loading control (N=3).

B. Representative ERG trace of 1 day old flies expressing RDGB$^{(DDHD-LNS2)\Delta}$ and relevant controls. Y-axis represents amplitude in mV, X-axis represents time in sec.

C. Representative images of fluorescent deep pseudopupil from 1 day old flies of expressing RDGB$^{(DDHD-LNS2)\Delta}$ and relevant controls expressing the PH-PLCδ::GFP probe.

D. Western blot of protein extracts made from fly heads expressing RDGB$^{(DDHD-LNS2)\Delta}$ and relevant controls and the PH-PLCδ::GFP probe. The blot is probed with antibody to GFP. Syntaxin is used as a loading control (N=3).

E. Western blot of protein extracts made from fly heads of RDGB$^{LNS2\Delta}$ and relevant controls. The blot is probed with antibody to RDGB. Tubulin is used as a loading control (N=3).

F. Representative ERG trace of 1 day old flies expressing RDGB$^{LNS2\Delta}$ and relevant controls. Y-axis represents amplitude in mV, X-axis represents time in sec.

G. Representative images of fluorescent deep pseudopupil from 1 day old flies of expressing RDGB$^{LNS2\Delta}$ and relevant controls along with the PH-PLCδ::GFP probe.

H. Western blot of protein extracts made from fly heads of RDGB$^{LNS2\Delta}$ and relevant controls, expressing PH-PLCδ::GFP probe. The blot is probed with antibody to GFP. Syntaxin A1 is used as a loading control (N=3).

Biology Open • Supplementary information

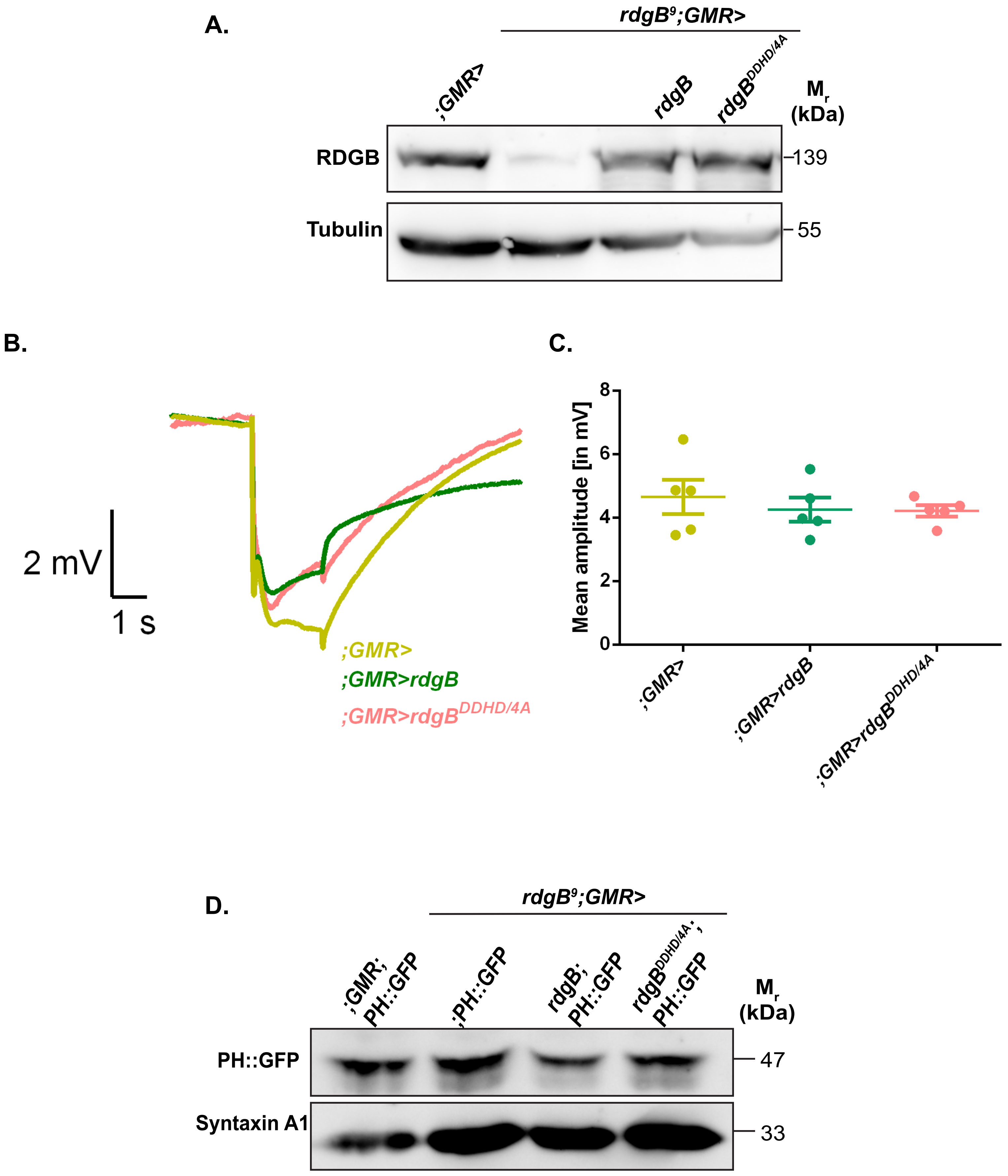

Basak et al., Supplemental Data 3

Biology Open • Supplementary information

Figure S3.

A. Western blot of protein extracts made from fly heads of $RDGB^{DDHD/4A}$ and relevant controls. The blot is probed with antibody to RDGB. Tubulin is used as a loading control (N=3).

B. Representative ERG traces from 1 day old flies expressing $RDGB^{DDHD/4A}$ and the relevant controls. Y-axis represents ERG amplitude in mV, X-axis represents time in sec. Genotypes studied are indicated.

C. Quantification of the light response from 1 day old flies expressing $RDGB^{DDHD/4A}$ and controls. Each point on Y-axis represents mean amplitude ±s.e.m., n=5 flies per genotype (ANOVA followed by Tukey's multiple comparison test, error bars indicate s.e.m.).

D. Western blot of protein extracts made from fly heads of $RDGB^{DDHD/4A}$ and relevant controls expressing PH-PLCδ::GFP probe. The blot is probed with antibody to GFP. Syntaxin A1 is used as a loading control (N=3).

Biology Open • Supplementary information

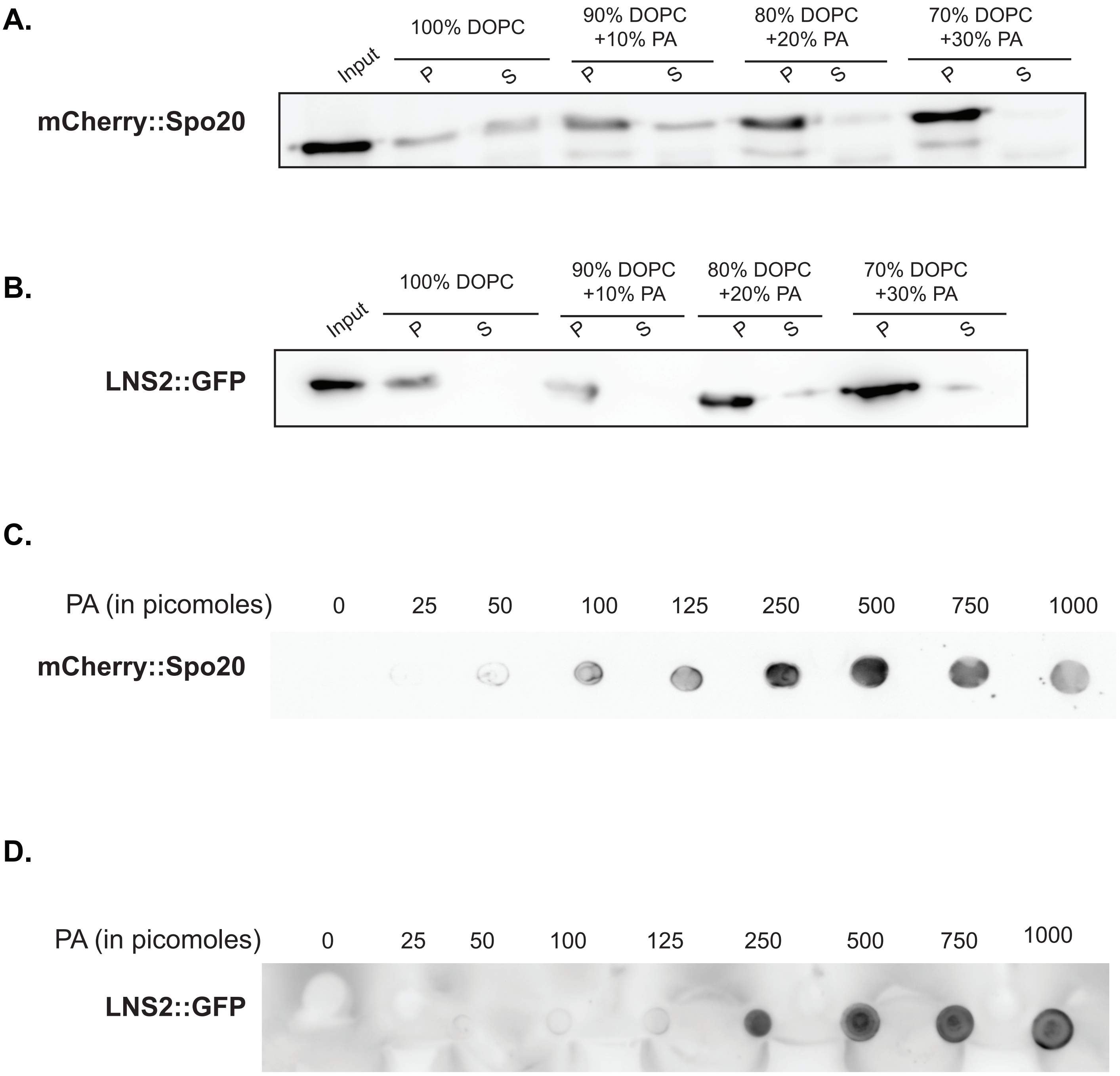

Basak et al., Supplemental Data 4

Figure S4.

A. Western blot showing the binding of mCherry::Spo20, a positive control for PA binding, to liposomes with increasing concentration of PA. The blot is probed with antibody to mCherry (P= pellet fraction, S= supernatant).

B. Western blot showing the binding of LNS2::GFP to liposomes with increasing concentration of PA. The blot is probed with antibody to GFP (P= pellet fraction, S= supernatant).

C. Nitrocellulose membranes spotted with increasing picomoles of PA were incubated over night with S2R+ cell lysate expressing mCherry::Spo20, a positive control for PA binding. Binding is detected by probing with anti-mCherry antibody.

D. Nitrocellulose membranes spotted with increasing picomoles of PA were incubated over night with S2R+ cell lysate expressing LNS2::GFP. Binding is detected by probing with anti-GFP antibody.

Biology Open • Supplementary information

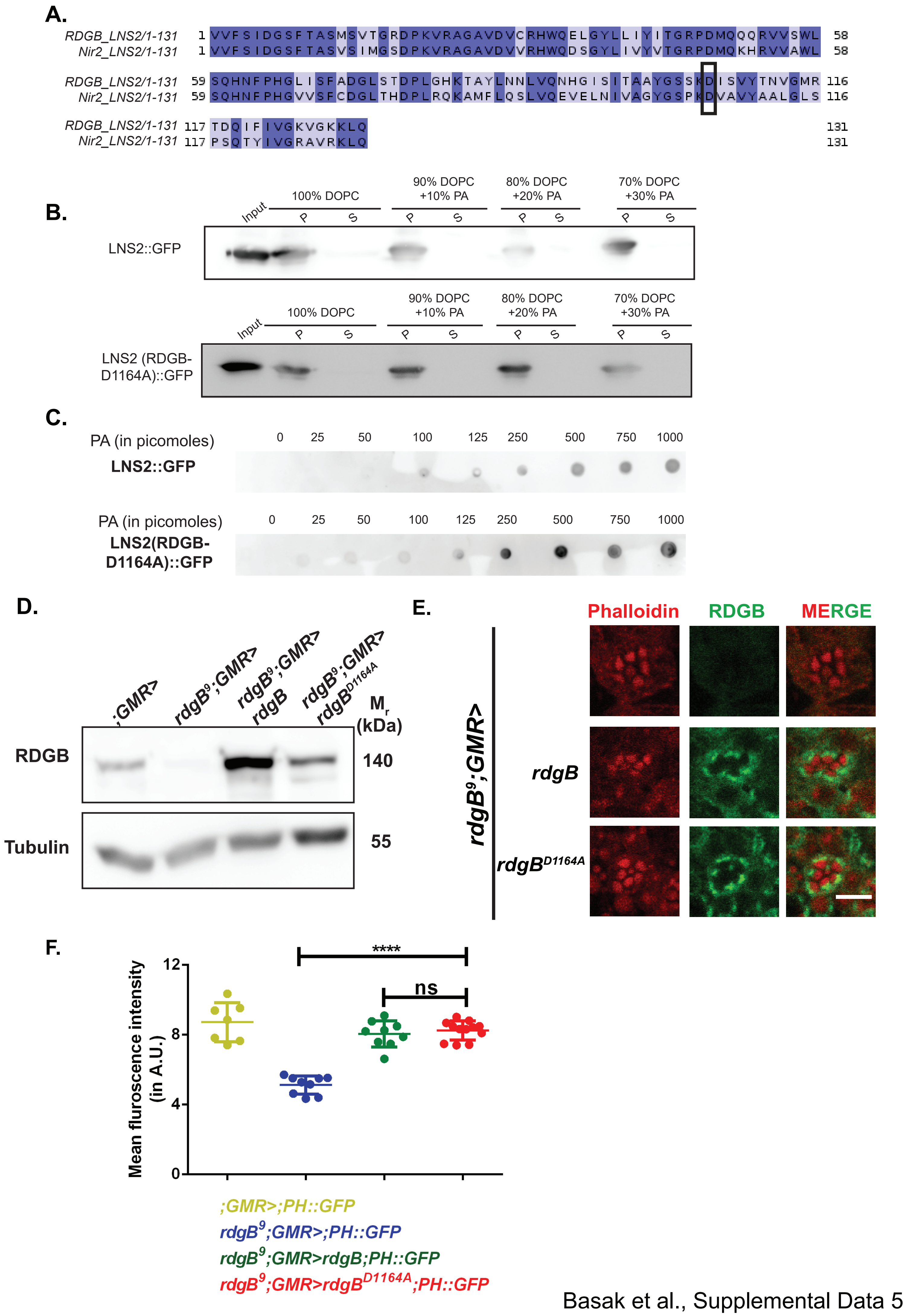

Basak et al., Supplemental Data 5

Biology Open • Supplementary information

Figure S5.

A. The alignment between the LNS2 domain of RDGB and Nir2 is generated using clustalO. The alignment is colour coded using JalView at sequence percent identity of 40% and above. The shades of blue represent identity between 40-100%.

B. Western blots showing the binding of LNS2::GFP and LNS2 (RDGB-D1164A)::GFP to liposomes with increasing concentration of PA. The blots are probed with antibody to GFP (P= pellet fraction, S= supernatant).

C. Nitrocellulose membranes spotted with increasing picomoles of PA were incubated over night with S2R+ cell lysate expressing LNS2::GFP and LNS2(RDGB-D1164A)::GFP for control. Binding is detected by probing with anti-GFP antibody.

D. Western blot of protein extracts made from fly heads of $RDGB^{D1164A}$ and relevant controls. The blot is probed with antibody to RDGB. Tubulin is used as a loading control (N=3).

E. Confocal images of retinae obtained from flies expressing $RDGB^{D1164A}$ and controls. Transverse sections of an individual ommatidium are shown. Red represents phalloidin which marks the rhabdomeres and green represents immunostaining for the RDGB protein. Scale bar= 5 µm.

F. Quantification of the fluorescence intensity of the deep pseudopupil from flies expressing $RDGB^{D1164A}$ and controls. Y-axis denotes the mean intensity per unit area (A.U. =Arbitrary Units) ±s.e.m., n>=7 flies per genotype (ANOVA followed by Tukey's multiple comparison test, error bars indicate s.e.m.**)**

Biology Open • Supplementary information
